# Biomechanical Behavior of Endodontically Treated Abutment Teeth with Periapical Lesions: A 3D Finite Element Analysis

**DOI:** 10.1055/s-0045-1814774

**Published:** 2026-02-05

**Authors:** Ömer Kırmalı, Ayten Zeynallı, Türker Akar, Huseyin Kursat Celik

**Affiliations:** 1 Department of Prosthodontics, Faculty of Dentistry, Akdeniz University, Antalya, Türkiye; 2 Private Practice, Dinçalya Oral and Dental Health Polyclinic, Antalya, Türkiye; 3 Department of Prosthodontics, Faculty of Dentistry, Erzincan University, Erzincan, Türkiye; 4 Department of Agricultural Machinery and Technology Engineering, Akdeniz University, Antalya, Türkiye

**Keywords:** finite element analysis, periapical lesion, root canal treatment, stress distribution, fixed partial denture

## Abstract

**Objectives:**

The stress distribution of teeth with periapical lesions, which are used as abutments in fixed prostheses, and its effect on the success of the restoration are not well known. This study investigates stress distribution in abutment teeth with periapical lesions and surrounding tissues under occlusal load using finite element analysis.

**Materials and Methods:**

Three models were constructed: (1) a healthy mandibular premolar, (2) a premolar with a periapical lesion restored using a fiber post and single crown, and (3) a similar premolar used as an abutment for a three-unit bridge. A static load of 300 N was applied at a 45-degree angle to the tooth's long axis, targeting the lingual slope of the buccal cusp. Stress and deformation were analyzed across all structural components, including dental tissues and the surrounding trabecular bone.

**Results:**

The results demonstrated that, in model 2, stress propagation extended more prominently along the root surface and progressed apically, resulting in a higher stress concentration in the periapical lesion region (0.061 MPa) when compared with model 3 (0.054 MPa). Furthermore, the greatest deformation was also observed in model 2. Across all models, peak stress was consistently localized in the cervicobuccal collar region of the tooth.

**Conclusion:**

These findings underline the importance of prosthesis design in reducing the stress concentration in abutment teeth with periapical lesions and demonstrate the biomechanical advantage of splinted restorations over single crowns in cases with periapical lesions.

## Introduction

Modern dentistry primarily aims to treat patients who lost dental function and phonation, to provide oral esthetics, and to promote oral health for various reasons. Dental function decreases after endodontic treatment in decayed, eroded, and traumatized teeth with advanced substance loss, indicating a need for methods to restore such teeth. 1

The type of restoration and root canal treatment (RCT) can affect the prognosis of teeth with periapical lesions. 2 Ongoing research studies aim to find new materials that can withstand intraoral forces while also providing esthetics like dental tissue. Material selection, the geometry of the support tooth preparation, and connector thickness are crucial for, particularly in bridge restorations, reducing the stress on the abutment teeth and providing sufficient resistance. 3

Although vital teeth are preferred, teeth with RCT and periapical lesions can also be used as an abutment in fixed prosthetic applications. Most of the periapical lesions are treated with appropriate endodontic treatment. 4 Healing occurs within 6 to 12 months of RCT. 5 Postponing the placement of the coronal restoration during the healing period results in an increased risk of tooth fracture. Placing a solid coronal restoration positively affects periapical healing. 6 In this type of tooth, post-core systems made by taking support from the root canal are used to provide retention to the coronal restoration. 7 Post materials with material properties similar to dentine reduce stress and increase resistance to fracture as they transfer the incoming forces to the root efficiently. 8 Studies show the survival rate of endodontically treated teeth restored with fiber posts and crowns is 85.1% after 10 years of use, which is six times greater than that of teeth restored without crowns. 9

Standardization of all factors and evaluation of a single factor are difficult and expensive in in vivo studies. For this reason, engineering simulations of experimental biomechanical studies in dentistry are being conducted with the finite element analysis (FEA) due to its reliability and ease of calculation advantages. Numerical methods-based computer codes play an important role in determining clinical and biomechanical states through the calculation of stress, strain, and deformations. 10 The success of restorative dental procedures depends significantly on the stress distribution within the tooth and surrounding tissues. Teeth with periapical lesions present a unique challenge due to altered stress distribution, which can impact the long-term success of restorations. 11 12 FEA provides a valuable tool for evaluating these stress patterns. This study aims to investigate the stress distribution in root canal treated premolar teeth with periapical lesions used as abutments in fixed partial dentures with use of FEA. In the literature, the number of studies investigating the stress distribution occurring in the periapical lesion and the surrounding bone tissue in prosthetic restorations using such teeth is limited. The null hypothesis is that the presence of a periapical lesion does not significantly alter the biomechanical stress distribution in root canal treated premolar teeth when used as abutments in fixed prosthesis.

## Materials and Methods

This study was planned to evaluate how the presence of a periapical lesion affects the stress distribution of the material, using the FEA on mandibular premolar tooth models that served as an abutment for a bridge. All solid models used in the analyses were created by remodeling the dental components separately in SolidWorks (SolidWorks Corp., Massachusetts, United States) three-dimensional (3D) parametric solid modeling software taking as a reference the real-size 3D human tooth models published by the School of Dentistry, University of Dundee. A reverse engineering approach was utilized in the remodeling of the tooth's subcomponents. The reverse engineering approach reproduces an object with the aid of drawings, documentation, or computer model data. 13 The 3D FEA examined how the presence/absence of a periapical lesion in the tooth affects the behavior of tooth structures under load, due to the stress occurring in a mandibular premolar tooth. This tooth served as the abutment for crowns and bridges.

RCT and subsequent postrestoration was simulated using the 3D computer-aided design model. ANSYS Workbench (ANSYS, Canonsburg, United States) finite element (FE) code was employed for the structural analysis. Following general assumptions were applied: (1) the geometry of the restored mandibular premolar tooth and prosthetic components was idealized to reduce unnecessary surface irregularities while preserving clinically relevant 3D features, (2) the cementum layer was considered as a separate part of dentin, (3) it was assumed that there was no gap between components and all components were perfectly bonded, (4) fixed supports were assigned to the basal and lateral bone surfaces to prevent rigid-body motion, while the remaining external surfaces were left unconstrained, (5) prestresses due to RCT were not taken into account, and (6) all materials were considered homogeneous, linear, and isotropic. A periapical lesion-free reference model was also created to examine the effect of different parameters on the mechanical performance of the restoration. The dentin component was planned to be created in conjunction with post, core, crown, periodontal ligament (PDL), and gutta-percha. Since 4 mm of gutta-percha was left clinically in the apical area in the model created after RCT and before post-placement, the length of the remaining gutta-percha in this area was determined as 4 mm, and the distance between gutta-percha and apical termination was 0.5 mm. The thickness of PDL was planned as 0.2 mm (on average). Related components were modeled as bone (cortical and trabecular), PDL, root, gutta-percha, post, post cement, core, crown cement, and crown. The model with a glass fiber post (ParaPost Fiber White, Coltène/Whaledent, Mahwah, New Jersey, United States) was created with a diameter of 1.5 mm and a length of 15 mm. The post was designed to be parallel and tapered toward the end. The diameter of the ferrule was determined as 1.5 mm, and the thickness of the cementum was approximately 0.25 mm, considering the entire root as dentin. Thicknesses of dual-cure resin cement (Ivoclar Vivadent, Schaan, Liechtenstein) between dentin-post and crown-core were determined as 0.1 and 0.2 mm, respectively. In core restoration, a 135-degree chamfer-style step ending at the gingival level was created using resin composite (ParaPost Fiber White, Coltène/Whaledent). In the crown restoration, metal supported porcelain crown was applied, and a premolar tooth model was obtained. After the periapical lesion-free reference model was created, a total of three clinical models were created to compare the mechanical behavior against the load. The periapical lesion was modeled as a spherical void with a diameter of 4 mm at the apex of the root, and this geometry was standardized across all models to ensure consistency. The first model was a healthy tooth; this model did not include a post and was used as a reference condition for interpreting the mechanical influence of post-restored scenarios. The second model was a single-body crown mandibular premolar tooth with a periapical lesion, and the third model was a three-member bridge abutment mandibular premolar tooth with periapical lesion.

**Fig. 1:**
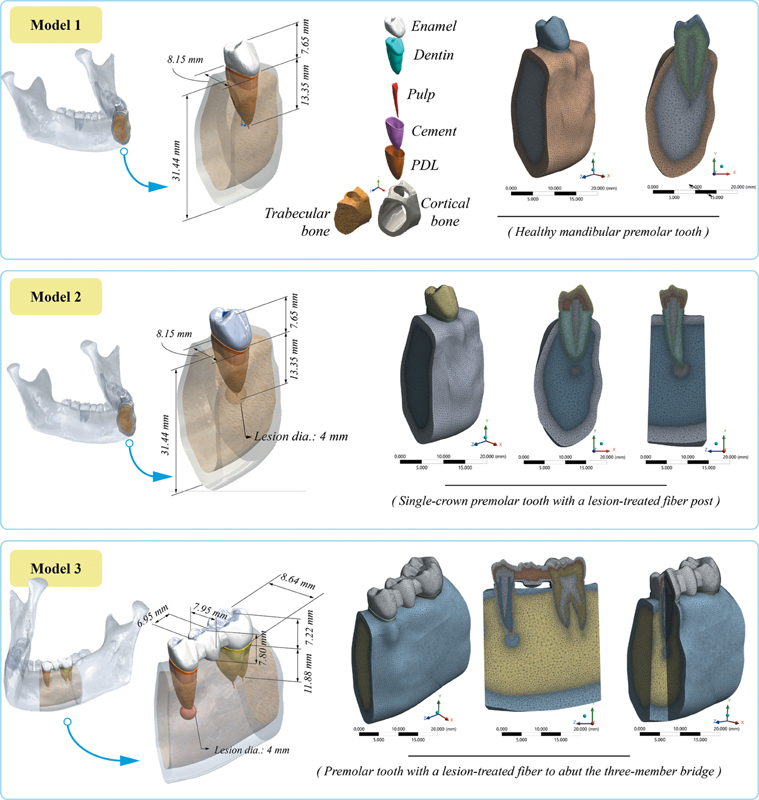
Mesh structure of the models utilized in the finite element analysis (FEA) scenarios.

The mesh structure (FE model) was created using the ANSYS Workbench meshing interface. Smaller element sizes were used for the contact surfaces to best describe the model's geometries, creating the highest quality contact relations. Visualization of the mesh structure is given in Fig. 1 .

The FE model was created with incremental element sizing-based meshing. Tetrahedral elements with a growth rate of 1.2 and a minimum size of 0.2 mm were used. Here, while the maximum element size was 2.5 mm in noncritical regions, the mesh was refined to 0.2 mm at the root, post, and lesion interfaces to improve accuracy and capture stress gradients effectively. The skewness metric was employed in the internal validation of the mesh structure. One of the primary quality measures for a mesh structure in a FEA is the skewness metric, which measures the shape and asymmetry of a distribution, 14 indicating how close to ideal a face or cell is in a FE model. According to the definition of skewness in the analysis code, a value of zero (0) indicates an equilateral cell (best) and a value of one (1) indicates a completely degenerate cell (worst). 15 FE models with FE quality with an average skewness metric value of 0.235 were obtained ( Table 1 ).

**Table 1:** FE model details

Details of the FE models (mesh structure)	
Job title	Dental FEA
Meshing approach	No adaptive sizing
Average skewness value/quality measure	0.235 ± 0.005/Excellent a
Element types (ANSYS WB Code)	Tet10
Max. element size (mm)	2.5
Min. element size (mm)	0.2
Defeature size (mm)	5.00E-03
Element growth rate	1.2
Total number of elements	1731855 ± 507768 a
Total number of nodes	2562638 ± 768938 a

Abbreviations: FE, finite element; FEA, finite element analysis.

a Average value for three FEA setups.

Material properties describing their physical and mechanical properties were assigned to each of the tooth components that make up the models. The homogenous isotropic linear elastic material model assumption was considered in this study. The material properties' elastic modulus, Poisson's ratio, and density were assigned to the FEA code based on previous research studies ( Table 2 ). 10 16 17 18 19 20

**Table 2:** Properties of materials assigned in the FEA scenarios

Material	Modulus of elasticity (MPa)	Poisson's ratio (-)	Density (kg m ^-3^ )
Enamel	84100	0.33	2800
Dentin	18600	0.31	2200
Cortical bone	14000	0.323	1400
Cancellous bone	1370	0.38	850
Pulp	2	0.45	1000
Periodontal ligament (PDL)	68.90	0.45	1100
Cement	8200	0.31	2030
Lesion tissue	0.69	0.45	370
Glass fiber post	37000	0.27	2500
Composite	12400	0.3	2400
Crown (porcelain)	68900	0.28	2300
Gutta-percha	140	0.45	370
Adhesive resin cement	7600	0.3	2000
Substructure (Ni-Cr)	204000	0.3	7600

Abbreviation: FEA, finite element analysis.

a FEA definitions: Homogenous isotropic linear elastic material model.

When previous related studies were evaluated in the literature, it was observed that the occlusal static load on each of the mandibular premolar tooth models was applied at a value of 300 N and an angle of 45 degrees to the long axis of the tooth on the lingual inclination of the buccal apex of the tooth. In this study, the preferred occlusal static load was determined to be similar to the studies. 10 21 22 23 24

To solve the analyses in the created FEA scenarios, the surface relations of the components (contact conditions) of the model were introduced to the FEA code. In the study, it was assumed that all components were exactly in contact with each other, which was defined as bonded contact in the FEA code. This contact definition was identically applied to models 2 and 3 to maintain consistency in component separation and interaction across all analyses.

## Results

This study provides new insights into stress distribution in abutment teeth with periapical lesions, providing quantitative evidence to inform clinical decisions and bridge the gap between digital modeling and restorative practice. When a total of three analysis scenarios were examined, it was observed that after occlusal load applications, stress was concentrated in similar regions, but its intensity varied; in model 1, significant stress concentration was observed around the buccal margin, whereas in model 2, stress was found to be more prominent in the cervical and middle thirds of the root. Numerical outputs for the maximum deformation and stress magnitudes by components are given in Table 3 .

**Table 3:** FEA numerical results

Output		Components	FEA study code		
			Model 1	Model 2	Model 3
Max. deformation	(mm)	Global (whole model)	0.091	0.098	0.082
Maximum von Mises stress values	(MPa)	Global (whole model)	123.300	133.870	130.610
		M. cortical bone	117.020	86.925	60.086
		M. trabecular (cancellous) bone	15.405	14.682	9.604
		Crown (porcelain / bridge / enamel)	123.300	109.200	86.595
		Substructure (Ni-Cr)	–	133.870	130.610
		Lesion	–	0.061	0.054
		Fiber post	–	31.515	20.592
		Dentin	48.539	47.214	37.517
		Cement	65.939	47.494	23.248

Abbreviation: FEA, finite element analysis.

**Fig. 2:**
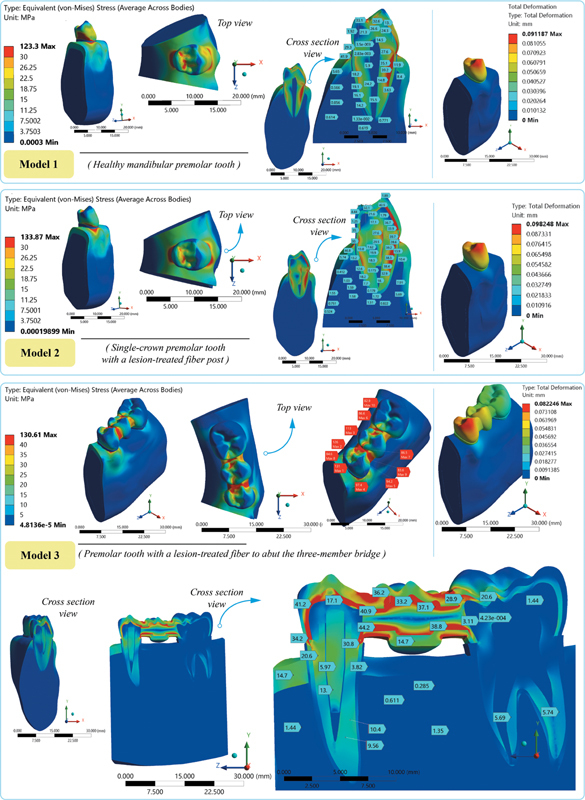
Finite element analysis (FEA) visual results.

In all models, a decrease in stresses on the alveolar bone and tooth root was observed descending from the cervical line to the apex ( Fig. 2 ). This result suggests that there is a more effective load transfer or distribution mechanism further away from the point where the force is first applied. The stress distribution was not homogeneous due to the direction of applied force and tooth anatomy. Model 2 exhibited higher stress in the periapical lesion area (0.061 MPa) compared with model 3 (0.054 MPa), as the stress spread more along the root in model 2 ( Fig. 1 ). The higher maximum deformation observed in model 2 (0.098 mm) compared with the lower deformation of model 3 (0.082 mm) is consistent with a more diffuse stress distribution across the root and periapical lesion.

**Fig. 3:**
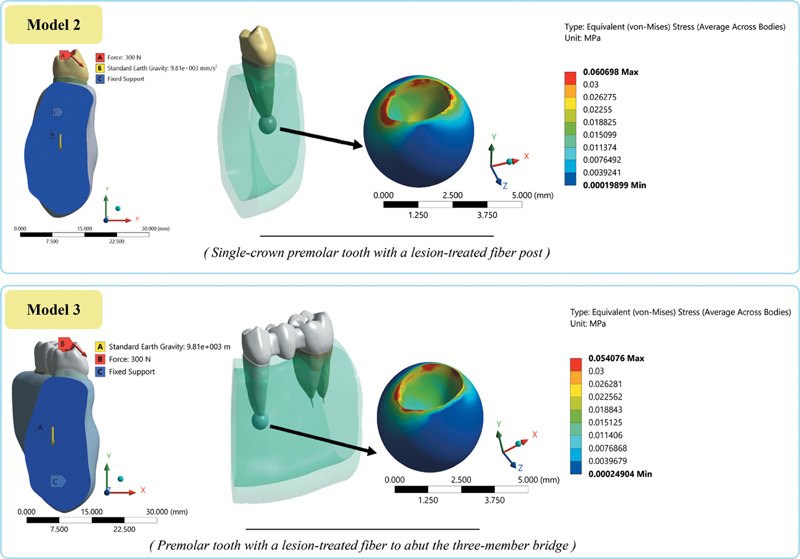
Stress distributions in the periapical region in model 2 and model 3.

In all the models, maximum stresses were seen in the cortical bone. While the lowest value was observed in model 3 (60.08 MPa), the highest value was detected in model 1 (117.02 MPa). Model 3 had a homogeneous stress distribution (indicating efficient load transfer across the bridge structure) at the ceramic-metal pontic connection; however, the stress was more prominent on the entire buccal root in model 2 (86.92 MPa) ( Fig. 3 , models 2 and 3). However, the stress also concentrated considerably at the coronal region in crowned model (model 2). The total deformation distribution was observed in higher values in model 2 like model 1 (0.091 mm), in comparison to model 3, that displayed the lowest values.

## Discussion

The study hypothesized that there would be no significant difference in stress distribution between using a tooth with a periapical lesion as a single crown abutment (model 2) and as a bridge abutment (model 3). Based on these results, the null hypothesis, which states that the presence of a periapical lesion does not significantly alter the biomechanical stress distribution in root canal treated premolar teeth when used as abutments in fixed prosthesis, is rejected. The results demonstrated that the presence of a periapical lesion increased both stress and deformation, as observed when comparing model 2 (lesion, single crown) with model 1 (healthy, no lesion). Moreover, when two lesion-bearing conditions were compared, higher stress and deformation values were obtained in model 2 than in model 3 (lesion, bridge), indicating that the splinted bridge design provided a more favorable biomechanical response by distributing the occlusal load more uniformly along the root and supporting bone.

FEA method, which is used to evaluate the stress caused by chewing force in prosthetic treatments, has advantages, but also disadvantages and limitations. Although linear elastic stress analysis is generally accepted in dentistry, the deformation occurring in tissues and materials may not be directly proportional to the force. 25 26 27 28 In addition, any of the materials found in the nature is not considered to be completely homogeneous and isotropic, dental materials are considered homogeneous and isotropic in studies. The material values were considered homogeneous and isotropic as the mean value due to these properties, which can change depending on many factors. This affected the results of the study. 26 Therefore, in the present study, the structures were accepted as homogeneous, isotropic, and linear. These simplifications, while common in FEA studies, may lead to an underestimation or overestimation of actual stress values, particularly in biological tissues that exhibit nonlinear and anisotropic behavior. Therefore, the numerical results presented in this study should be interpreted within the context of these accepted FEA assumptions.

In the current study, in all models, stresses were concentrated at the level of the enamel-cement junction in the buccal collar area of the crown, as shown in Fig. 3 . This agrees with the findings of Gomes et al 28 who observed stress concentration in areas with high modulus of elasticity, emphasizing the importance of material properties in load distribution. Shetty et al 29 investigated the effect of esthetic post systems on stress distribution and determined that in the model created by applying a glass-fiber post system, the stress in the dentin was concentrated in the cervical and middle third of the root. In another FEA study, conducted by Coelho et al 18 on the maxillary central incisors restored with post and full ceramic crown, for all post systems, including the tooth model restored with glass fiber post, the stress distribution in dentin was concentrated in the buccal and palatal regions, especially in the middle third of the root. Compared with other post types, the stress distribution in the fiber post-applied model was more homogeneous for all tooth structures. Due to the differences in the use of model 2 as a crown and model 3 as a bridge restoration abutment, the stress distribution in the fiber post in model 2 was found to be higher than in model 3. Similar to the study 18 in both models, more stress distribution was observed in the middle third of the fiber post, on the surface suitable for the cervicobuccal area of the tooth ( Fig. 3 ). Toksavul et al 30 concluded that the stress in dentin for all models, including the glass fiber post, was concentrated in the buccal and coronal thirds of the root surface as a result of their study on the effect of stress distribution of different post types. In the present study, the stress distribution in the dentin was concentrated in the buccal collar region of the tooth. These values were similar in model 1 and model 2, but lower for model 3, which has a bridge abutment that distributes the load over a larger area.

Belli et al 26 conducted a study on the effect of root canal filling on stress distribution in mandibular premolars with endodontic-periodontal lesions. There was no significant difference in stress distribution between the tooth model with only primary endodontic lesion and the endodontically treated tooth model restored using various canal filling materials; whereas, in other models, in which periodontal diseases were added, a significant increase was observed in stress. Maximum stresses had the highest value in the force-applied buccal cusp. Similar to the results of this study, in the current study, there was no significant difference between the values of the models with periapical lesions (model 2, model 3) and in the lesion area with low stress and total deformation distribution. The maximum stresses had the highest value in the buccal collar region. This result indicates that the type of restoration minimally affects the stress at the lesion area.

In another study, Belli et al 31 examined root canal-filled mandibular premolars restored with post and all-ceramic crown and simulated the bone tissue surrounding the models, primary endodontic, primary endodontic secondary periodontal involvement, and real combined lesions through FEA. In the primary endodontic lesion model, it was accepted that the periapical lesion diameter was 2 mm and it was observed that the fiber post (with an elastic modulus similar to dentin) showed homogeneous stress distribution and stress increased when compared with the tooth model without lesions. It was concluded that although there was a lesion in the periapical part of the tooth, the glass fiber post could still preserve the root structure due to its elastic properties similar to dentin. In this study, similar data were obtained, and the stress was low and homogeneous in the core structure and root, but at lower values than dentin in both models containing fiber post. The stress distribution in the fiber post in model 2 was higher than in model 3. In both models, more stress distribution was observed in the middle third of the fiber post, on the surface suitable for the cervicobuccal area of the tooth.

Kırmalı et al 32 simulated a total of seven models, including sound, root canal treated, ankylosis, and crowned third molars in their FEA study, in which they examined the stress distribution in autotransplanted molars. They observed that occlusal morphology and the presence of PDL affected stress distribution in dentin and cortical bone. Higher stresses were observed in trabecular bone in teeth with normal periodontal space and in cortical bone in ankylosed teeth. In the ankylosed model, stress was observed primarily in the coronal region of the tooth. Stress concentration in the cervical region and possible fractures in ankylosed teeth can be prevented by crowning the tooth. In this study, a decrease was observed in the stresses in the alveolar bone and tooth root as the tooth descended from the cervical to the apical in all models. Although this situation was not homogeneous depending on the application direction of force and tooth anatomy, a significant decrease was remarkable. The stress values observed in cortical and trabecular bone in teeth with lesion were lower than the healthy tooth model. Similarly, in this study, the highest cortical bone stress observed in model 1 (117,020 MPa) reflects direct load transfer in a healthy, unrestored tooth, while the progressive decrease recorded in model 2 (86,925 MPa) and model 3 (60,086 MPa) is attributed to the influence of restorative treatments and the presence of a periapical lesion on load distribution.

As the number of fixed prosthesis members increases, the amount of stress that occurs on the abutments under occlusal forces also increases. The rate of deformation varies as much as the cube of the number of pontic in both prosthetic and fixed prosthetic restorations on the implant depending on the number of pontics. For example, if all other conditions remain the same, the number of deformations increases by eight times in bridges with two pontics, and 27 times in bridges with three pontics.

In our study, when the lesioned tooth was used as a bridge abutment, stress distributions in all components were observed at lower values compared with a single crowned tooth. This finding is in agreement with the observations of Chitumalla et al 33 that bridges affect stress distribution even in abutment teeth with loss of periodontal attachment, indicating that bridged restorations can distribute occlusal loads more effectively even in lesioned teeth. In the present study, stress concentration was observed in the buccal collar, buccal tubercle, and connector regions of the premolars that were subjected to bridge forces. In particularly, there is a higher stress distribution in the connector in the distal region of the premolar tooth, compared with the mesial connector of the molar tooth ( Fig. 3 ). This is consistent with the findings of Onodera et al 34 that fractures in Y-TZP prostheses and Wimmer et al 35 that fractures in polymethyl methacrylate prostheses usually occur at the distal connector, emphasizing that the connector regions are critical for high stress concentration.

Our findings of stress concentration in the connector regions are supported by Almasi et al 36 who showed in their FEA study that the cross-sectional shape and area of the connector affect the load distribution. The connectors with elliptical cross-sections distribute the stress more homogeneously, demonstrating the importance of these structural features for optimal bridge design. The findings for the bridge models in the present study are in general agreement with the FEA study of Karakurt 37 using different superstructure materials (metal-backed ceramic, monolithic zirconia, etc.) and force directions (vertical/inclined). In their study, it was observed that the maximum stress values were concentrated in the crown collar or connector regions depending on the material and force direction, confirming that the biomechanical behavior of bridge restorations depends on the material selection and design details.

In summary, the results of this study consistently showed that the stress distribution values by FEA were significantly higher in model 2 (single crown abutment) compared with model 3 (bridge abutment). This important finding underlines the biomechanical advantage offered by fixed partial dentures: In model 3, the occlusal force applied to the premolar was effectively distributed across the pontic through the connector. This load-sharing mechanism naturally reduced local stress concentrations on the abutment tooth, especially in the presence of a periapical lesion, and offered a potentially more favorable biomechanical environment

The main limitation of this study is that mathematical models were used to imitate clinical conditions. In addition, the tooth geometry was intentionally idealized with smooth internal and external surfaces, without anatomical grooves or root depressions, to ensure standardization across models. This simplification may have produced a distinct junction between the gutta-percha and the post that appears as a local stress concentration in the FEA results; however, this feature reflects geometric modeling rather than a clinical defect. In the digital environment, it is not possible to model the structures examined in FEA studies exactly as they are in the natural environment. The forces occurring in an oral environment have both static and dynamic structures, and the thermal transitions resulting from oral activities are not the same in the mathematical model. While the anatomical structures of the modeled teeth are not the same in every human being, ideal tooth forms and structures have been standardized in these studies. For this reason, it is necessary to support the data of the present study with clinical studies and to carry out long-term in vivo clinical follow-ups that examine the possible effects on the underlying tissues. In addition, according to the results of the present study, research topics such as increasing the number of teeth with lesion to be used as abutments, supporting different prosthetic restorations, and examining the effect of occlusal forces with different morphologies and different prosthetic materials arose. The results of this study were observed under force applied to the premolar tooth at an angle of 45 degrees; therefore, other studies can investigate how the combined (vertical and oblique) forces applied to the teeth during chewing will affect the teeth with lesion. This study did not simulate surgical endodontic procedures (e.g., apicoectomy); therefore, our conclusions pertain to nonsurgically managed teeth with periapical lesions, and the suitability of an apicoectomized tooth as a bridge abutment should be determined case by case after documented periapical healing and confirmation of adequate root and periodontal support.

The clinical implication of this study is that the biomechanical response of endodontically treated premolars with periapical lesions is markedly influenced by the restorative design. The analysis showed that single-crown restorations produce higher stress concentrations at the cervical and apical regions, whereas splinting the same tooth within a three-unit bridge provides more uniform load transfer and lower stress magnitudes along the root and supporting bone. These findings indicate that, in clinical situations involving periapical pathology, bridge-supported restorations may offer a biomechanically more favorable and prognostically safer option than single crowns. 7 31

## Conclusion

Within the limitations of this FEA study, the following conclusions were drawn:

1. The presence of a periapical lesion and the type of restoration both influenced the stress distribution along the root surface. Compared with the healthy tooth (model 1), the lesion-bearing models exhibited altered stress transfer patterns, while the splinted bridge configuration (model 3) showed lower overall stresses than the single-crown restoration (model 2).
2. In both lesion-bearing models (model 2 and model 3), stress and total deformation within the lesion region were low, but the bridge-supported design provided a more uniform and biomechanically favorable distribution of stresses along the root and surrounding bone.
